# Epidemiology of Treated Attention-Deficit/Hyperactivity Disorder (ADHD) across the Lifespan in Taiwan: A Nationwide Population-Based Longitudinal Study

**DOI:** 10.1371/journal.pone.0095014

**Published:** 2014-04-15

**Authors:** Charles Lung-Cheng Huang, Chin-Chen Chu, Tain-Junn Cheng, Shih-Feng Weng

**Affiliations:** 1 Department of Psychiatry, Chi Mei Medical Center, Tainan, Taiwan; 2 School of Medicine, Kaohsiung Medical University, Kaohsiung, Taiwan; 3 Department of Anesthesiology, Chi Mei Medical Center, Tainan, Taiwan; 4 Department of Recreation and Health-Care Management, Chia-Nan University of Pharmacy and Science, Tainan, Taiwan; 5 Department of Neurology and Occupational Medicine, Chi Mei Medical Center, Tainan, Taiwan; 6 Department of Occupational Safety, College of Environment, Chia Nan University of Pharmacy and Science, Tainan, Taiwan; 7 Department of Occupational and Environmental Medicine, National Cheng Kung University Hospital, Tainan, Taiwan; 8 Department of Medical Research, Chi Mei Medical Center, Tainan, Taiwan; 9 Department of Hospital and Health Care Administration, Chia Nan University of Pharmacy and Science, Tainan, Taiwan; University of Akron, United States of America

## Abstract

**Objectives:**

We used insurance claims of a nationally representative population-based cohort to assess the longitudinal treated prevalence and incidence of attention-deficit/hyperactivity disorder (ADHD) in children, adolescents and adults.

**Methods:**

Participants were identified from among National Health Insurance enrollees in Taiwan from 1999 to 2005. We identified study subjects who had at least one service claim during these years with a principal diagnosis of ADHD. A total of 6,173 patients were recorded in the treated ADHD cohort during the 6-year study.

**Results:**

There was a significant increase in the treated prevalence rate of ADHD during the study period, from 64.65 per 100,000 in 2000 to 145.40 per 100,000 in 2005 (*p* = .001). An increase in the treated incidence rate of ADHD, from 44.67 per 100,000 in 2000 to 81.20 per 100,000 in 2005, was also observed (*p* = .013). However, the treated prevalence of ADHD was still lower than that of the community data in Taiwan. The peak treated prevalence of ADHD was at age 7–12 years for both males and females, and the peak treated incidence of ADHD was at age 0–6 for females and age 7–12 for males. Overall, the treated incidence and prevalence rates dropped abruptly after age 13–18 (both *p*<.001) for males and females (*p*<.001 for both). Male vs. female ratios of treated prevalence and incidence were both above 1 before age 25–30 years, but below 1 thereafter.

**Conclusion:**

Although an increasing number of people with ADHD sought treatment during 1999–2005 in Taiwan, the treated prevalence of ADHD was still lower than that of the community data. The treated incidence and prevalence of ADHD fell dramatically after age 13–18. However, more women than men sought treatment in adulthood. There may be under-diagnosis and under-treatment of ADHD, especially among females and adults.

## Introduction

Attention-deficit/hyperactivity disorder (ADHD) is characterized by behavioral symptoms of inattention, hyperactivity, and impulsivity across the life cycle and is associated with considerable morbidity and disability [1], [2]. Although it has long been recognized that ADHD is one of the most prevalent psychiatric disorders among children [3], the fact that this disorder often persists into adulthood and has a devastating impact has only recently become the focus of attention [4], [5], [6]. ADHD affects approximately 3% to 12% of school-aged children and adolescents [7], [8], [9], [10], [11], with a higher prevalence among males [7], [9], [10]. The symptoms may decline with age [12], [13], [14]. Nevertheless, the available data suggest that ADHD has a persistence rate of 15% to 78% into adulthood [4], [13], [15], depending on how one defines persistence. Epidemiology data on adult ADHD are limited, but several attempts have been made to gather this data and yielded prevalence estimates in the range of 1–6% [11], [14], [16], [17].

ADHD impacts several domains of the lives of those who suffer from its symptoms, their family, and society [5], [18], [19]. Patients with untreated ADHD are associated with poorer long-term self-esteem and social functioning outcomes than non-ADHD controls [20], [21]. Treatment for ADHD is associated with reduced core symptoms and improvement in outcomes [4], [20], [21]. However, it has been suggested that ADHD is underdiagnosed and undertreated [22], [23], especially in girls [24] as well as in adults [25], [26], [27]. In spite of the public health implications due to the high prevalence and considerable morbidity and disability in childhood and adulthood as a result of ADHD, only a few data exist on the treatment patterns of ADHD in routine clinical practice [25], [26], [28], and knowledge regarding the adult group remains sparse [25], [26]. Take Taiwan for example, where only one previous study known to us has estimated the epidemiology of treated ADHD in children aged 1–8 years [29]. This study reported a cumulative incidence up to age 8 of 2.69% for ADHD in 2004. There is evidence that factors such as genetic disposition, neurochemical abnormalities, neuropsychological deregulation, and environmental factors persist across the lifespan or change age-dependently [6], [30]. Nevertheless, basic information on how the treated prevalence and incidence of ADHD varies by age and gender remains poorly described. Another gap in the current ADHD literature is that, although there have been both cross-sectional [26], [31] and longitudinal epidemiological estimates [28], [29] of treated ADHD, few studies [25] have explored the longitudinal trends of ADHD prevalence and incidence across the lifespan, using nationwide datasets. To our knowledge, only one study has examined the prevalence and incidence trends of pharmacologically treated ADHD in children, adolescents, and adults [25]. The authors found that a trend of increasing prescribing prevalence of ADHD drug treatment was observed within each age category from 2003 to 2008 in UK. Prevalence of prescribing to adult patients increased; however the numbers treated are much lower than published estimates of the prevalence of ADHD. The databank used in that study consisted of information provided by general practitioners in the UK, however. In an attempt to fill this gap, we designed a study to evaluate the annual incidence and prevalence of pharmacologically-treated ADHD (may include other nonpharmacological and multimodal management for adjuvant therapy) in healthcare-seeking children, adolescents, and adults among a nationally representative general population in Taiwan for the period 1999 to 2005. The inclusion of 6 years of data with a wide age range allowed us to investigate longitudinal trends of treated ADHD prevalence and incidence across the lifespan.

## Methods

### Sample

In this study, the medical claims data were retrieved from the Taiwan’s National Health Insurance Research Database (NHIRD). The confidentiality of individuals was protected using encrypted personal identification to avoid the possibility of the ethical violations related to the data. Exemption was obtained from the institutional review board of Chi Mei Medical Center. The study sample was taken from the database of medical claims, including outpatient (ambulatory) care, hospital inpatient care, dental services, and prescription drugs provided by the National Health Research Institutes (NHRI) in Taiwan for use in related healthcare service studies. The Longitudinal Health Insurance Database 2005 is a sub-dataset of the NHIRD that contains all claims data (from 1996 to 2011) of one million beneficiaries who were randomly selected in 2005. The data consists of outpatient (ambulatory) care and inpatient care records and the registration files of the Taiwan NHIRD; as many as 93.1% of the residents of Taiwan in 1999 and 99.2% in 2009 were enrolled in the national health insurance system. There were no statistically significant differences in age and sex between the sample group and all enrollees.

### The Definition of ADHD

Using the International Classification of Disease, 9th Revision, Clinical Modification (ICD-9-CM) diagnostic criteria [2], study subjects were identified as those who had at least one service claim (either outpatient or inpatient care) with a principal diagnosis of ADHD (ICD-9-CM: 314.XX) between 1999 and 2005.

In Taiwan, most of ADHD patients are diagnosed and treated by psychiatrists. They are diagnosed through clinical interview, scale assessment, and neuropsychological test (e.g. attention ability) in either outpatient or inpatient care. The physicians register the diagnosis code of the patient to the national health insurance system according to the ICD-9-CM diagnostic criteria. The treatment is pharmacotherapy mostly, but may also include nonpharmacological and multimodal management for adjuvant therapy, such as psychoeducation, counseling, behavioral intervention, and family therapy.

### The Treated Prevalence of ADHD

Study subjects described above were defined as the prevalent cases from 1999 to 2005. The treated prevalence of ADHD from 1999 to 2005 was also calculated. The treated prevalence of ADHD was calculated with the number of total study subjects at the beginning of each year as the denominator and the number of prevalent cases of ADHD in each year as the numerator.

### The Treated Incidence (Newly Diagnosed) of ADHD

The study subjects who were defined as having ADHD during the year and had not been defined as having ADHD during the previous years were defined as incident cases. The treated incidence of ADHD for each year was calculated from 1999 to 2005. The denominator was the person-years that were contributed by the study subjects. The study subjects who were alive at the end of the year contributed one person-year to the denominator. The study subjects who died during the year contributed one-half person-year to the denominator. The numerator was the number of incident cases.

### Measurements and Statistical Analysis

We obtained the information on age and sex directly from the Bureau of National Health Insurance (BNHI)’s insured file. Age was categorized into one of 8 groups comprising every 6 years from 0 to 48 years (e.g., 0–6, 7–12, etc.), and older than 49 years. The trend analyses of years were tested by linear regression using overall 6-year treated incidence and prevalence of ADHD. The Chow test [32], a particular test for structural change to determine whether the coefficients in a regression model are the same in separate sub-samples, was used to specify the breakpoint (e.g., age 13–18 years) in terms of the position of the observation.

The SAS statistical package SAS System for Windows, version 9.3.1 (SAS Institute Inc, Cary, North Carolina, USA) was used to perform the analyses in this study.

## Results

A total of 6,173 patients were enrolled in the treated (healthcare-seeking) ADHD cohort during the 6-year study.

**Figure 1:**
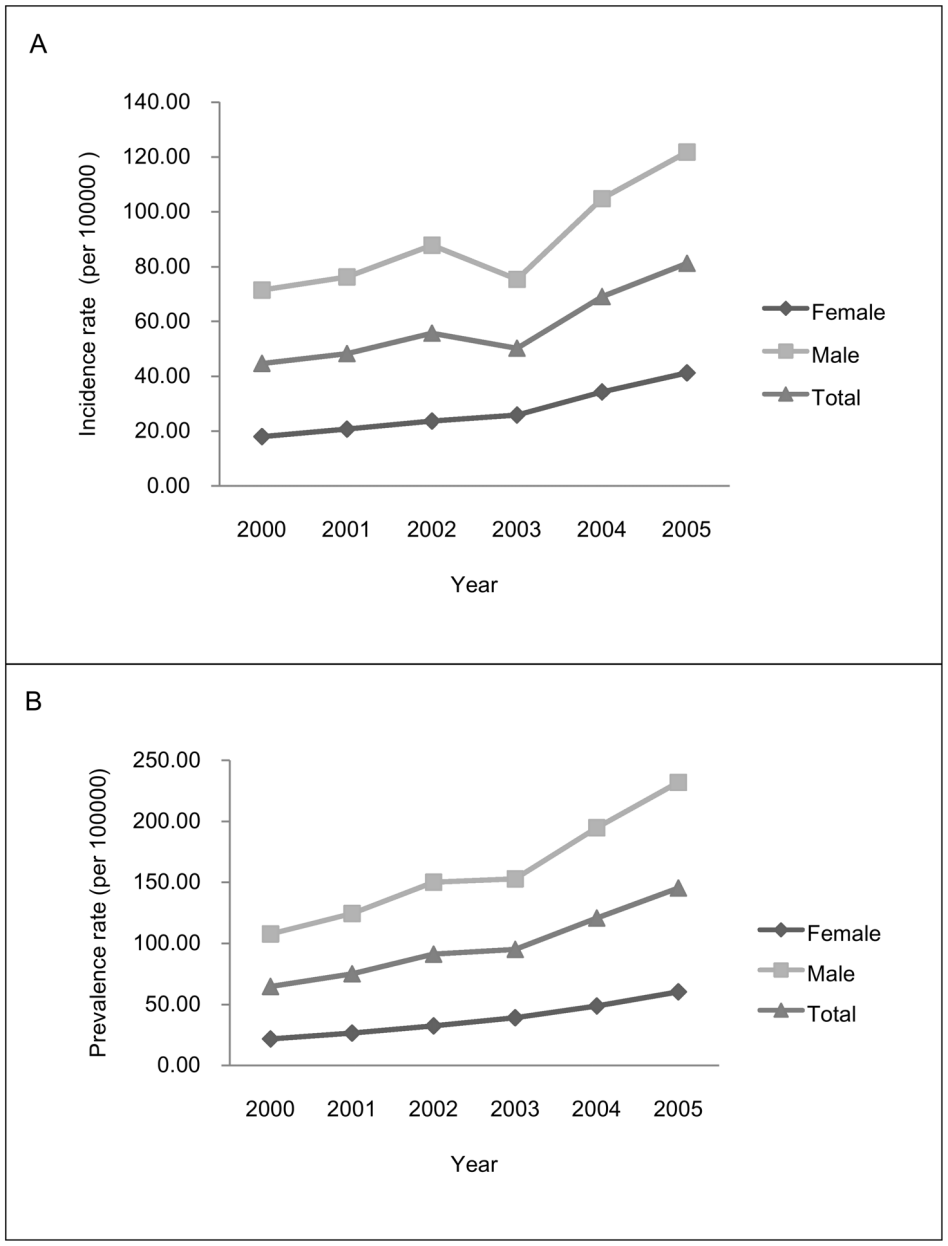
Overall 6-year healthcare-seeking incidence and prevalence of ADHD by years. (A) The trend analyses of year tested by linear regression were women (beta = 4.55; P = 0.002), men (beta = 9.29; P = 0.023), and total (beta = 6.85; P = 0.013). (B) The trend analyses of year tested by linear regression were women (beta = 7.61; P<0.001), men (beta = 23.85; P = 0.001), and total (beta = 15.56; P = 0.001).

There was a significant increase in the prevalence rate of treated ADHD using linear regression for trend analyses of years, from 64.65 per 100,000 in 2000 to 145.40 per 100,000 in 2005 (beta = 15.56, *p* = .001). Increasing trends were found for both males (beta = 23.85, *p* = .001) and females (beta = 7.61, *p*<.001). There was a trend toward an increase in the incidence rate of treated ADHD, from 44.67 per 100,000 in 2000 to 81.20 per 100,000 in 2005 (beta = 6.85, *p* = .013), although there was a decrease in 2003. The treated prevalence and incidence rates of ADHD were both higher in males than in females for every calendar year. However, decreasing trends were noticed for the male-to-female ratio of treated incidence and prevalence during 2000–2005, ranging from 3.98 to 2.95 and 4.97 to 3.85, respectively (Table 1, Figure 1).

**Table 1:** Incidence rate and prevalence rate of ADHD in Taiwan, 1999∼2005.

	N (Incidence rate per 100,000 per year)			N (prevalence rate per 100,000 per year)			M/F ratio of incidence	M/F ratio of prevalence
	Female	Male	Total	Female	Male	Total		
1999				101 (22.16)	420 (93.83 )	521 (57.67 )		4.23
2000	82(17.97)	325(71.46 )	407 (44.67)	99 (21.70)	490 (107.74)	589 (64.65)	3.98	4.97
2001	97 (20.73 )	351 (76.23)	448 (48.26)	124 (26.50)	573 (124.44)	697 (75.08)	3.68	4.70
2002	111 (23.69 )	410 (87.82 )	521 (55.70)	152 (32.45)	701 (150.14)	853 (91.19)	3.71	4.63
2003	125 (25.86 )	353 (75.36)	478 (50.22)	189 (39.10)	716 (152.85)	905 (95.08)	2.91	3.91
2004	166 (34.27 )	494 (104.80 )	660 (69.06)	236 (48.72)	918 (194.76)	1154 (120.75)	3.06	4.00
2005	208 (41.25 )	604 (121.82)	812 (81.20 )	304 (60.30 )	1150 (231.94)	1454 (145.40)	2.95	3.85
Total	789	2537	3326	1205	4968	6173		

**Figure 2:**
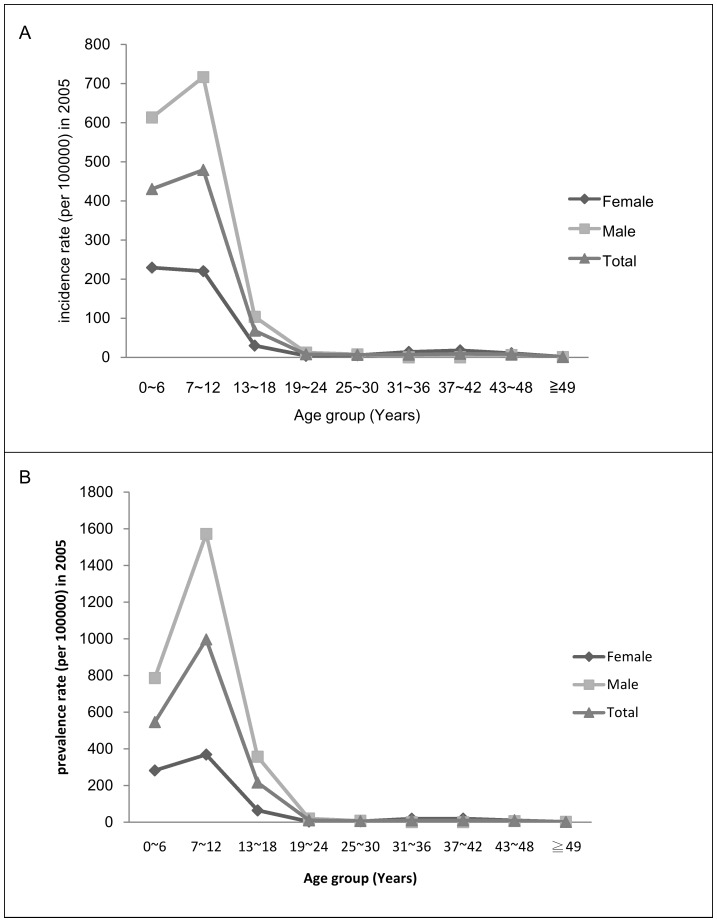
Healthcare-seeking incidence and prevalence of ADHD by age stratification in 2005. (A) Chow test (breakpoint age 13∼18) in women (F = 168.64; P<0.001), men (F = 146.24; P<0.001), and total (F = 167.34; P<0.001). (B) Chow test (breakpoint age13∼18) in women (F = 85.19; P<0.001), men (F = 43.48; P<0.001), and total (F = 48.49; P<0.001).

In 2005, the treated prevalence of ADHD was 231.94 per 100,000 for males and 60.30 per 100,000 for females, and the treated incidence was 121.82 per 100,000 for males and 41.25 per 100,000 for females. The peak treated prevalence of ADHD was in the 7–12 years age group for both males and females, and the peak treated incidence was at age 0–6 years for females and 7–12 years for males. Overall, the treated incidence rate dropped abruptly after age 13–18 years (F = 168.64, *p*<.001) for both males and females (both *p*<.001) using Chow test. The treated prevalence rate also dropped abruptly after age 16–18 years (F = 48.49, *p*<.001), for both males and females (both *p*<.001) (Table 2, Figure 2).

**Table 2:** Age- and sex-specific incidence (newly diagnosed) of ADHD by age stratification in 2005.

	N (Incidence rate (per 100,000))			N (prevalence rate (per 100,000))			M/F ratio of incidence	M/F ratio of prevalence
	Female	Male	Total	Female	Male	Total		
0∼6	82 (229.48)	241 (613.47)	323 (430.56)	101 (282.65)	309 (786.56)	410 (546.54)	2.67	2.78
7∼12	86 (220.45)	305 (716.85)	391 (479.41)	144 (369.13)	669 (1572.38)	813 (996.84)	3.25	4.26
13∼18	12 (29.88)	45 (103.67)	57 (68.21)	26 (64.74)	155 (357.10)	181 (216.60)	3.47	5.52
19∼24	2 (4.00)	5 (12.33)	7 (7.73)	2 (4.00)	8 (19.72)	10 (11.04)	3.08	4.93
25∼30	3 (5.31)	4 (7.74)	7 (6.47)	3 (5.31)	4 (7.74)	7 (6.47)	1.46	1.46
31∼36	7 (13.74)	0 (0.00)	7 (7.03)	10 (19.63)	0 (0.00)	10 (10.05)	0.00	0.00
37∼42	9 (17.91)	0 (0.00)	9 (8.95)	10 (19.90)	0 (0.00)	10 (9.94)	0.00	0.00
43∼48	5 (10.45)	3 (6.24)	8 (8.34)	5 (10.45)	3 (6.24)	8 (8.34)	0.60	0.60
≧49	2 (1.50)	1 (0.76)	3 (1.13)	3 (2.24)	2 (1.52)	5 (1.89)	0.51	0.68

M/F ratio = male-to-female ratio.

**Figure 3:**
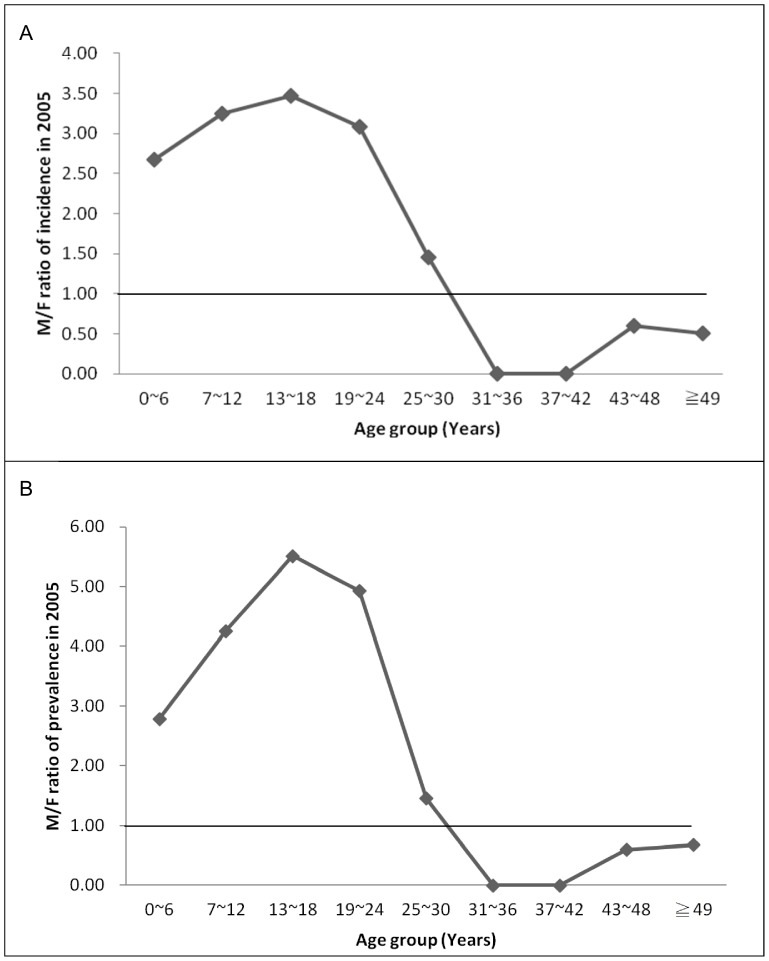
Male-to-female ratio of incidence and prevalence of ADHD by age stratification in 2005. M/F ratio = male-to-female ratio.

The overall male-to-female ratios of the treated prevalence and incidence of ADHD were 3.85 and 2.95, respectively. Male-to-female ratios of treated prevalence ranged from 1.46 to 5.52 before age 25–30 years, but fell below 1 thereafter. Male-to-female ratios of treated incidence ranged from 1.46 to 3.47 before age 25–30 years, but fell below 1 thereafter (Figure 3).

## Discussion

Our study is among the few that have examined healthcare-seeking ADHD patients across their lifespan, with a long observation period and a large sample of a general population, using data from Taiwan’s NHIRD. We designed a study to evaluate the annual incidence and prevalence of pharmacologically-treated ADHD in healthcare-seeking children, adolescents, and adults among a nationally representative general population in Taiwan for the period 1999 to 2005.

### Trends of Treated Prevalence and Incidence of ADHD

Overall, prevalence of treated ADHD increased during 2000–2005 from 64.65 per 100,000 to 145.40 per 100,000 patients. An increase in the treated incidence rate of ADHD was also observed, from 44.67 per 100,000 to 81.20 per 100,000. These findings are in line with figures reported in the literature, which show an increase in the prevalence of ADHD treatment during the past decade in the UK [25], the Netherlands [28], and the USA [33], [34]. Only one previous study known to us has estimated the epidemiology for treated ADHD in Taiwan [29]. The authors reported on the treated incidence in children aged 1–8 years from 1996–2004, and also found an elevated incidence rate for treated ADHD in later birth cohorts.

The results of this study suggest an increase in the prevalence and incidence of treated ADHD in Taiwan throughout the study period; however, the highest treated prevalence figure reported (15.7 per 1000 male patients aged 7–12 years) is below those reported in Germany (50 per 1000) [26], the Netherlands (21 per 1000) [28], and the USA (29 per 1000) [34], although is in line with the data from the UK (15.4 per 1000) [25]. It is worth noting that this number remains lower than the global prevalence of ADHD in community children and adolescents in Taiwan, which was estimated to be 9.9% and 3.3–7.5%, respectively [9], [10].

### Under-diagnosis and Under-treatment of Adult ADHD

Our data showed that the peak treated prevalence of ADHD was at age 7–12 years for both males and females. These findings are in line with figures reported in Western countries, including Germany [26], the UK [25], and the USA [34]. It was surprising that the treated incidence and prevalence rates for both males and females dropped abruptly after age 13–18 years. A recent study by McCarthy et al. used The Health Improvement Network (THIN) database to examine the epidemiology of pharmacological treatment in children, adolescents and adults between 2003 and 2008 in UK primary care [25]. They also demonstrated a dramatic fall in treated incidence and prevalence rates after age 13–17 years. Furthermore, the highest treated prevalence rate among adults in our study was 0.20 per 1000 male patients aged 19–24 years, which was much lower than the estimated figure for ADHD in the general population.

There may be several explanations for the under-treatment of ADHD patients in their adulthood. In Taiwan, Ritalin (methylphenidate) is the only stimulant that has been included in the benefit package of the National Health Insurance program for use in the treatment of ADHD in adults [35]. Concerta (prolonged-release methylphenidate) and Strattera (atomoxetine HCl) are only indicated as continuation treatment for adults who started their treatment with this medication in childhood or adolescence. The strict regulation may hamper the treatment of adult ADHD patients. Lack of awareness of this disorder among the general population and even clinical practitioners may be another important reason for the under-treatment of adults. A retrospective claims database analysis covering the insured population of Nordbaden in Germany also revealed that ADHD was rarely detected in the adult population [26]. In addition, most patients were not seen by a mental health specialist, and physician involvement was highly concentrated.

### Gender Effect on Treated Prevalence and Incidence of Healthcare-seeking ADHD

The present study has demonstrated that although the prevalence and incidence rates of treated ADHD were both higher in male patients for every calendar year, decreasing trends were noted for the male-to-female ratio throughout the study period, for both treated incidence and prevalence. These findings are in line with figures reported in the literature, which showed prevalence and incidence rates of drug prescribing were both consistently higher among boys than girls, although the greatest increases over time were observed in female patients [28], [33]. McCarthy et al. also found that while the proportions of prescribing prevalence for males were higher in the various age categories over time, the rates of increase in treated prevalence were greater in females than males, for patients aged 6–12 years, 13–17 years, and 25–45 years [25].

The general impression that male ADHD are much more likely to be clinically diagnosed and treated is mostly based on studies among children and adolescents [28], [33], rather than adult population. We found it interesting that the male to female ratios of treated prevalence and incidence were both above 1 before age 25 to 30 years, but below 1 thereafter. To our knowledge, a reverse male to female ratio of treated prevalence and incidence after adulthood has rarely been reported. Of the studies examining gender effects on the epidemiology of healthcare-seeking ADHD across the lifespan, one did not find a reverse ratio [26], and another found a reverse incidence ratio only among patients older than 45 years [25]. Since there was no evidence of a higher prevalence among females in the general population in the literature, there is a possibility that the higher treated incidence and prevalence among healthcare-seeking women in their adulthood may be associated with previously under-recognized ADHD. This finding echoes the argument of a previous study that ADHD may be under-identified in girls [24].

### Limitations and Strengths

This study has some limitations, however. The ADHD diagnoses used in the study were from administrative claims data reported by physicians, which may not be as precise as diagnoses made by structured interview with specific diagnostic criteria. Still another limitation of this study is that the sample size of adult patients was relatively small. Despite these considerations, the present study has important implications for practice and research. Our study is one of the few to explore longitudinal trends in the treated prevalence and incidence of healthcare-seeking ADHD patients across the lifespan, using nationwide datasets. The use of a nationwide population-based database such as this has advantages, such as (1) the large sample size; (2) preventing selection bias in recruiting study subjects; (3) avoiding the recall bias of patients when filling out questionnaires; (4) it may be more representative of the real world, i.e., allowing us to understand what is actually happening under the general conditions of clinical practice. This study has added to the existing body of knowledge on age and gender effects on the epidemiology of treated ADHD in clinical settings, in particular to the ADHD healthcare-seeking figures in adulthood.

### Conclusions

Epidemiologic data from Taiwan’s NHIRD in current study showed that although increasing numbers of people with ADHD had sought treatment during 1999–2005 in Taiwan, the treated prevalence of ADHD was still lower than in community data. The unique findings of this study were that there may be an under-diagnosis and under-treatment of ADHD, especially among females and adults. Nevertheless, more women than men sought treatment in their adulthood. This study has added to the existing body of knowledge on age and gender effects on the epidemiology of treated ADHD in clinical settings, in particular to the ADHD healthcare-seeking figures in adulthood. Furthermore epidemiologic studies can clarify whether the patterns of ADHD diagnosis and treatment in community and clinical settings is appropriate. Population-based epidemiologic studies may shed important new light on how we understand ADHD, as well as its natural history, treatment, and consequences.
